# Necrosis—A Strange Case

**Published:** 1874-11

**Authors:** E. M. Morrison


### NECROSIS—A STRANGE CASE.

BY E. M. MORRISON, M. D.

Master W., a lad of about fourteen years, in good health, came to my office, with his parents on the 13th of June, for treatment.

Upon examination, I found the case to be necrosis, but the history and cause were enveloped in obscurity and doubt so strangely, that the case is of much interest to me and may be worthy of consideration by others.

All that part of the alveolar process between the symphisis of the lower jaw and the center of the right lateral incisor was necrosed and detached, only retained in its place by its peculiar form and the closing of the adjacent teeth upon it from either side. The integuments and periosteum were gone, so that the dead bone was almost entirely denuded. By placing an elevator upon the lingual margin of the fragment, it was removed entire without difficulty as you see it, embracing the socket of the right central nearly to the apex of the root.

The tooth had been removed last February by a "dentist' of a neighboring town. After twenty-one efforts, according to the statement of the boy's mother, a good and truthful lady, the tooth was left remaining, but broken off at the margin of the gum and medicine applied to kill the nerve. I learned further from this lady that, after these Herculean surgical efforts and the application of this therapeutical skill, the remainder of the tooth, whose only offense was crowding, was removed without further difficulty in a few days.

What kind of medicine was applied could not be ascertained, but the fair presumption is that it was arsenic. Then, the question arises, was there any fracture of the bone at the time of the operation, or was the necrosis caused by the arsenic, independent of any fracture, and this portion of alveolar process removed by exfoliation?

On the one hand, the fracture of itself would not necessarily cause the death of the part, but that and the injudicious application of arsenic would be almost certain to produce this result. On the other hand, the unwarranted use of the arsenic and the probable injury to the periosteum would be almost certain to cause necrosis, and consequently exfoliation. Can we draw the line between fracture and exfoliation with sufficient distinctness to determine the question in all cases?

In general, this line is so very distinct that the question of diagnosis has not, so far as I know, been discussed in our standard text books on surgery. In fact, it is seldom that there is room for doubt, and there may not be in this case with those more skilled in diagnosis, but to the common observer the difference is not very apparent.

If the dentist was arraigned before a court of justice to answer for mal-practice, the history of the case might be developed, but this is not probable, and the only available witness at our command is the fragment of bone which has been removed. It would be a real pleasure, however, to be able to determine at once from this alone the question beyond a doubt. I am inclined to the theory of a fracture, but the time which has elapsed, four months, has been sufficient to cause great changes upon the surface and in the structure of the bone by the action of inflammation, and purulent and sanious discharges, so that the indications of fracture are not apparent to the unaided eye; that is we can not tell by the surface that it was fractured. The circumstance of violence also favors this view of the case, yet it might be a case of necrosis without the intervention of a fracture. The lower jaw is known to be quite liable to necrosis from various poisons, as phosphorus, mercury and arsenic, and the age of the patient would intensify this liability, so that the view favoring exfoliation is not without some plausibility.

Owing to these facts which so seldom come together and the moral and legal responsibility of the dentist, I have furnished you this case, together with the specimen, for your consideration, hoping that it will be thankfully received.

